# Supplementary material for: Tracing genetic diversity captures the molecular basis of misfolding disease
Source: Nat Commun. 2024 Apr 18;15:3333. doi: 10.1038/s41467-024-47520-0 (PMC11026414; doi:10.1038/s41467-024-47520-0)
Supplement: Supplementary file 1 — Supplementary Information [file 41467_2024_47520_MOESM1_ESM.pdf]

Supplementary information

Tracing Genetic Diversity Captures the Molecular Basis of Misfolding Disease

Authors: Pei Zhao[*], Chao Wang[*#], Shuhong Sun, Xi Wang, and William E. Balch[#]

*Contributed equally

#Address correspondence to: chaowang@szbl.ac.cn; webalch@scripps.edu

This supplementary information file includes

Supplementary Fig. 1-11

Supplementary Fig. 1

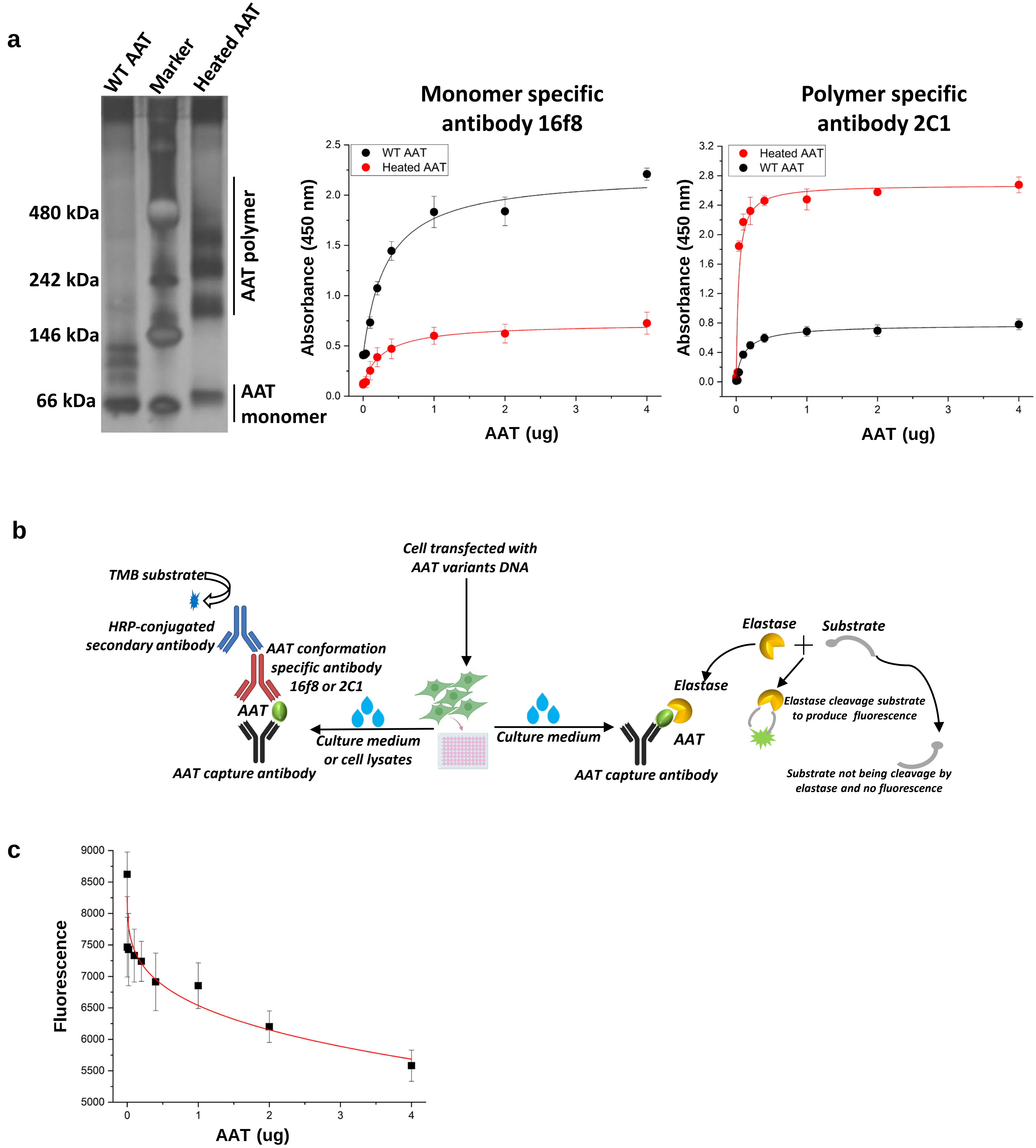

Supplementary Fig. 1. **Conformation dependent antibodies and high-throughput assays used to measure different AAT phenotypes**. (**a**) Shown is control WT monomeric and heat-treated polymeric AAT on native gel (left panel). Monoclonal antibody 16f8 generated shows strong interaction with WT monomeric AAT but not to heat-treated polymeric AAT (middle panel). Monoclonal antibody 2C1[52] shows a strong interaction responding to the heated polymeric AAT but not WT monomeric AAT (right panel). (**b**) A schematic figure showing the high-throughput assays used to measure the intracellular and secreted monomer or intracellular and secreted polymer pools using conformational dependent antibodies (see **Methods**). The activity of secreted AAT is determined by using a fluorogenic substrate of NE (see **Methods**). (**c**) The fluorescence of the NE substrate is dependent on the protein level of AAT. Data is presented as mean ± SD, n = 3 biologically independent measurements for absorbance or fluorescence signal.

Supplementary Fig. 2

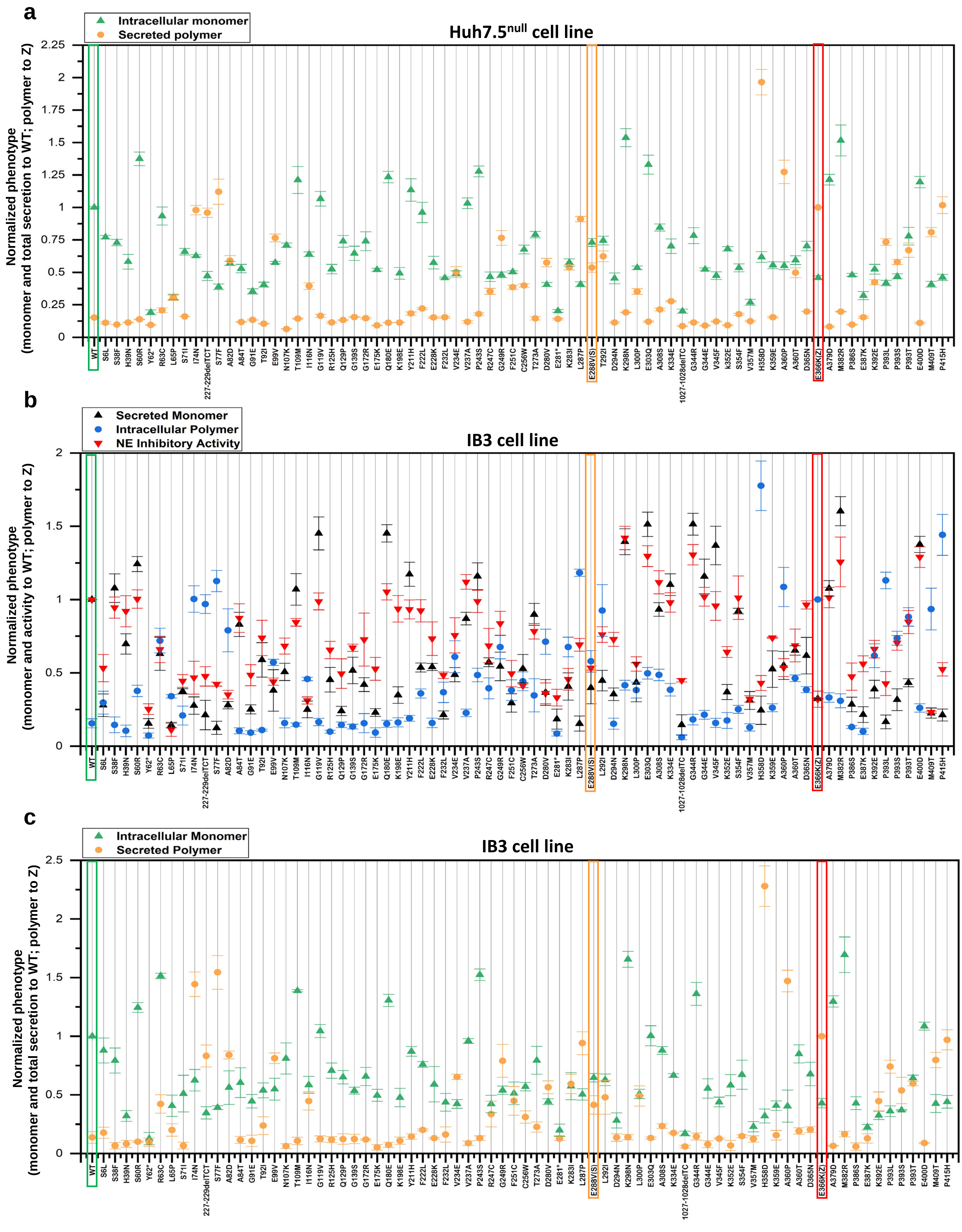

Supplementary Fig. 2. **Phenotypes of AAT variants in different cell lines**. (**a**) The intracellular monomer levels (blue) and secreted polymer levels (orange) of 75 AAT variants transiently expressed in Huh7.5^{null} cells (see **Methods**). (**b**) The secreted monomer levels, intracellular polymer levels and NE inhibitory activity for 75 AAT variants transiently expressed in IB3 cells. (**c**) The intracellular monomer levels and secreted polymer levels of AAT variants transiently expressed in IB3 cells. WT, E288V (S variant) and E366K (Z-variant) are highlighted. Data is presented as mean ± SD, n = 3 biologically independent measurements.

Supplementary Fig. 3

a

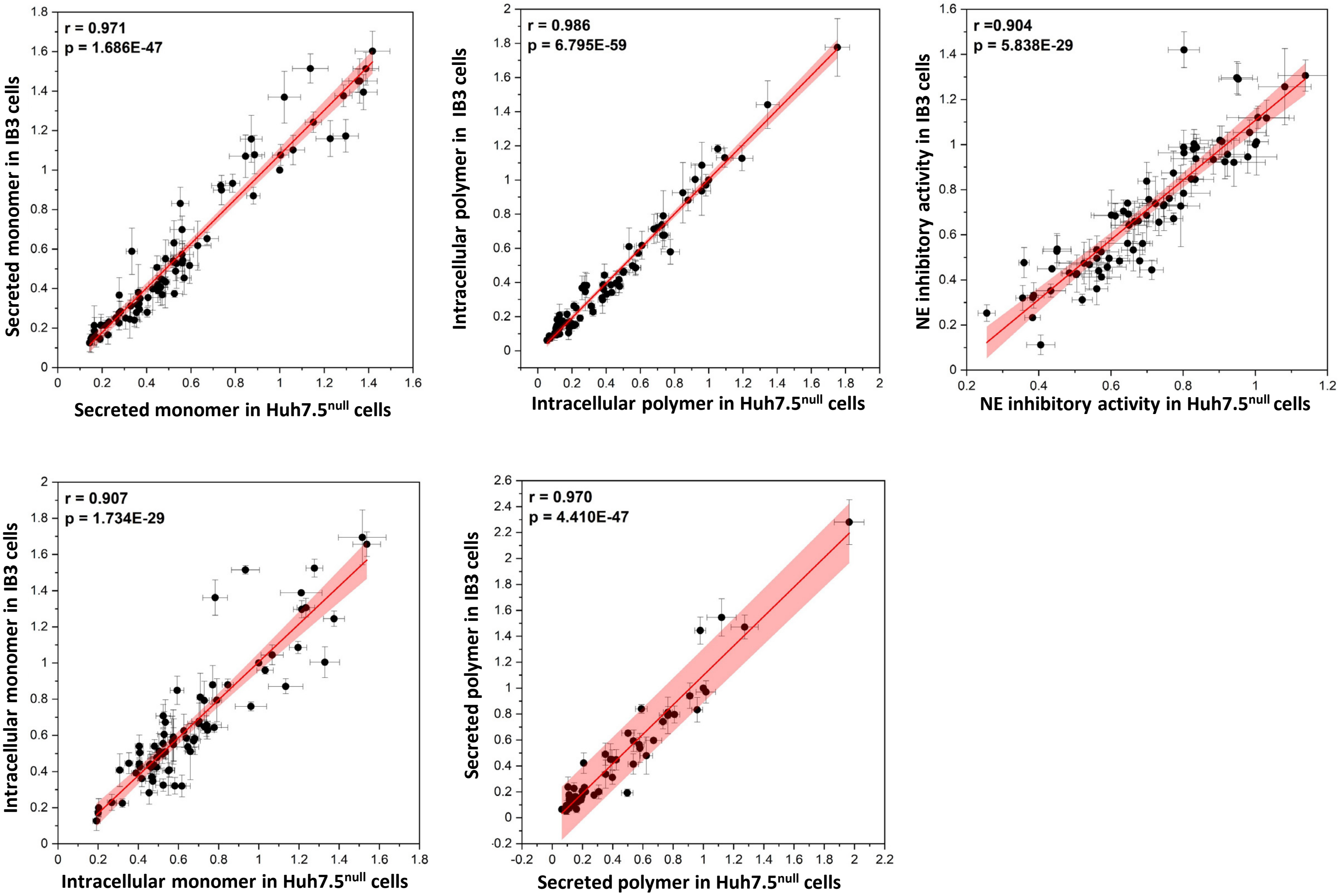

b

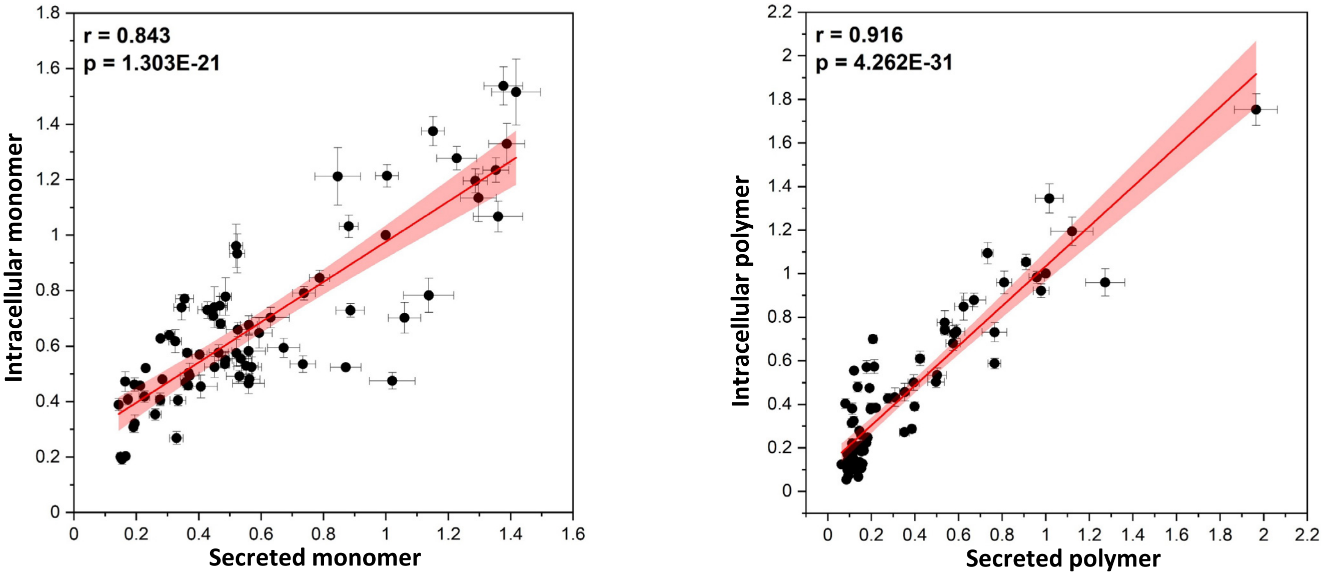

Supplementary Fig. 3. **Phenotype correlations between different cell types**. (**a**) The phenotype data of secreted monomer, intracellular polymer, NE inhibitory activity, intracellular monomer, secreted polymer for AAT variants are highly correlated between liver derived Huh7.5^{null} cells and lung derived IB3 cells. Person's r values and p values for each correlation are labeled. (**b**) In Huh7.5^{null} cells, shown is the correlation between intracellular monomer levels with secreted monomer levels of AAT variants (left panel) and the correlation between intracellular polymer levels with secreted polymer levels for AAT variants (right panel). Pearson's r values and p values for each correlation are labeled. Data is presented as mean ± SD, n = 3 biologically independent measurements.

Supplementary Fig. 4

a

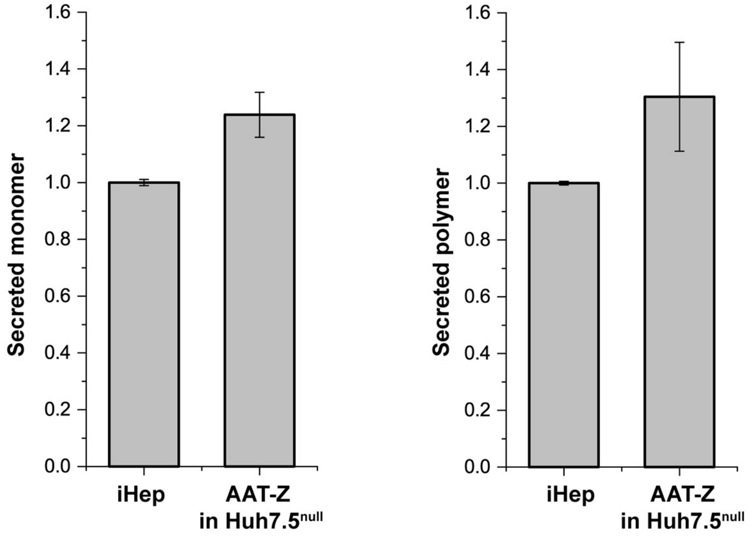

b

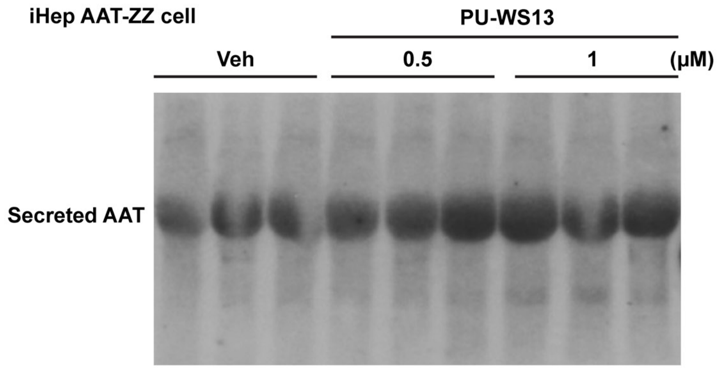

c

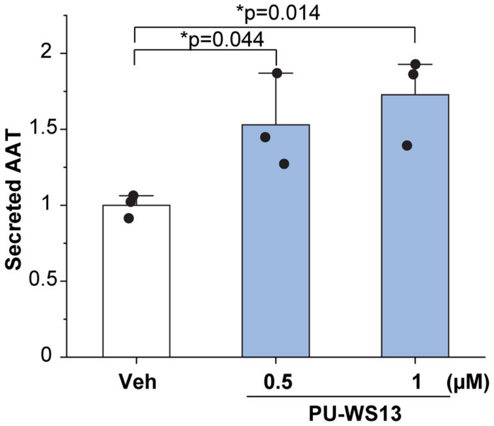

d

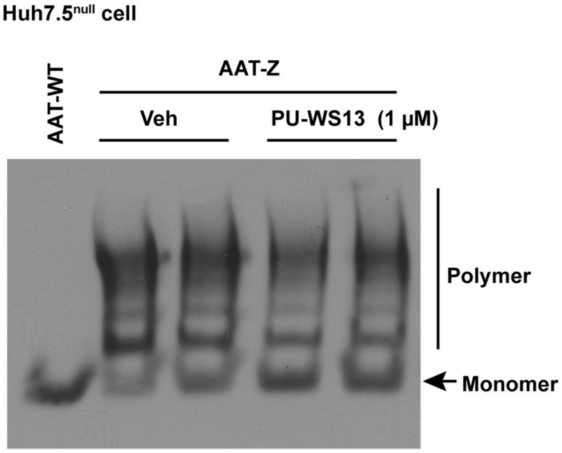

e

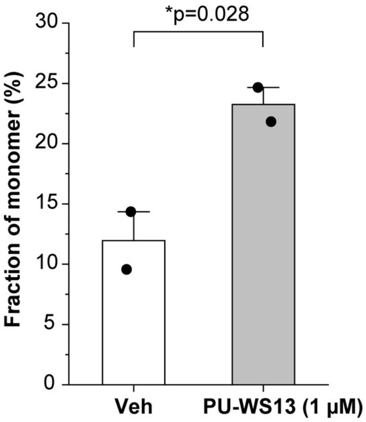

f

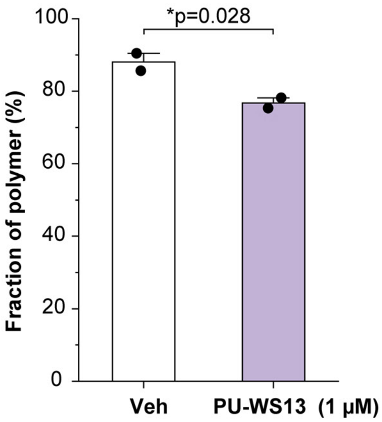

Supplementary Fig. 4. **PU-WS13 corrects AAT-Z in liver-derived cells. (a)** Secreted AAT-Z monomer and polymer from human iPSC-derived AAT-ZZ hepatocytes (iHepZZ cell) and Huh7^{null} cells transient transfected with AAT-Z[34]. Data is presented as mean ± SD, n = 3 biologically independent measurements. **(b-c)** Immunoblot analysis of the secretions from iHepZZ cell after the treatment of PU-WS13. iHepZZ cells were treated in presence or absence of PU-WS13 at 0.5 μM and 1 μM for 24 h. Culture medium was collected and subjected to Immunoblot analysis **(b)** and quantification **(c)**. Data is presented as mean ± SD, n = 3 biologically independent measurements. **(d)** Liver derived Huh7.5^{null} (AAT$^{-/-}$) cells transfected with AAT-Z plasmid were treated with PU-WS13 at 1 μM for 24 h. Culture medium was collected and analyzed by non-denaturing gel. **(e-f)** Quantification of the fraction of secreted AAT monomer (**e**) and polymer (**f**) from immunoblots as shown in (**d**). Data is presented as mean ± SD, n = 2 biologically independent measurements. (Student's t-test, two tailed for (**c**), one tailed for (**e-f**); *, $p<0.05$; **, $p<0.01$; ***, $p<0.001$; N.S., $p>0.05$).

Supplementary Fig. 5

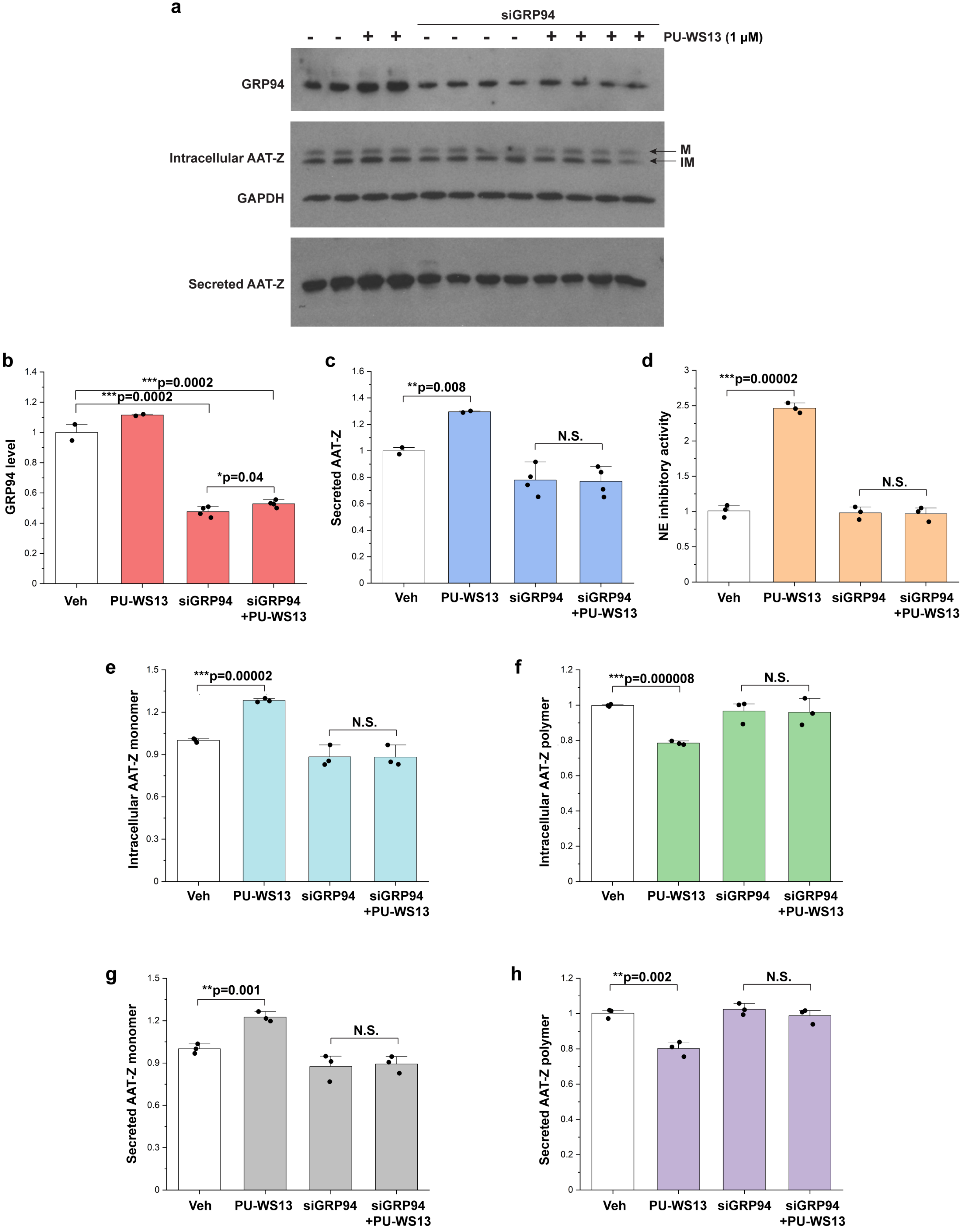

Supplementary Fig. 5. **The functional and conformational corrections of AAT-Z by PU-WS13 depend on GRP94.** AAT-Z stable expressed lung derived IB3 (AAT) cells were transfected with siRNA for GRP94 or no siRNA as vehicle control (Veh) for 48 h and then treated with PU-WS13 for 24 h **(a)**. Cellular GRP94 expression levels **(b)** and secreted AAT-Z (**c**) are quantified from immunoblot shown in **(a)**. The NE inhibitory activity of secreted AAT-Z proteins was measured using fluorogenic substrate of NE (**d**). Intracellular and secreted AAT-Z monomer was measured by ELISA using monomer specific antibody 16F8 (**e-f**). Intracellular and secreted polymer was measured by ELISA using polymer specific antibody 2C1 (**g-h**). Data is presented as mean ± SD, n=2 (Veh, PU-WS13), 4 (siGRP94, siGRP94+PU-WS13) from independent experimental samples. (Student's t-test, two tailed; *, $p<0.05$; **, $p<0.01$; ***, $p<0.001$; N.S., $p>0.05$).

Supplementary Fig. 6

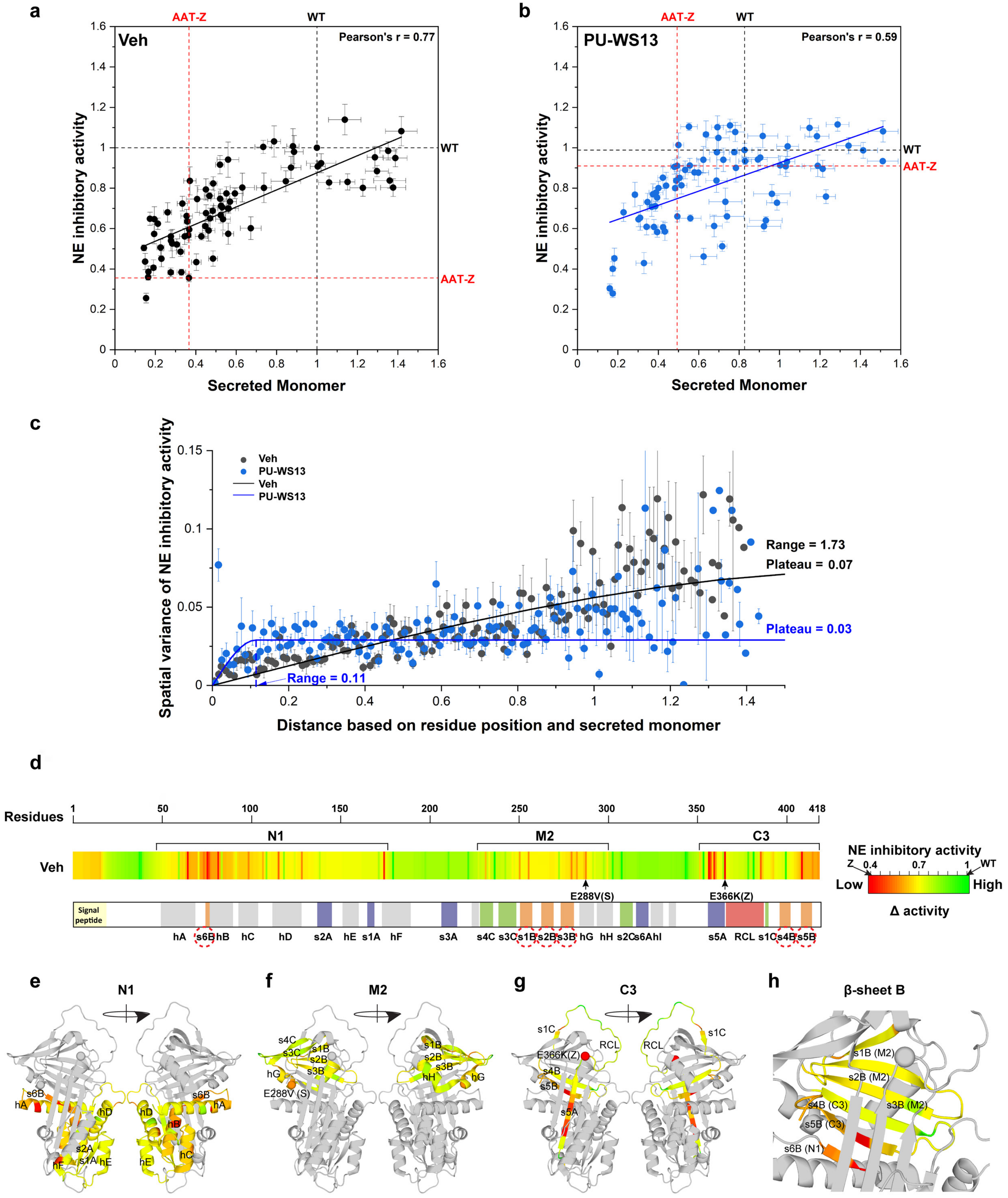

Supplementary Fig. 6. **PU-WS13 reshapes the relationships between monomer secretion and NE inhibitory activity. (a-b)** Linear correlation between secreted monomer and NE inhibitory activity of measured AAT variants in the absence **(a)** or presence **(b)** of PU-WS13. Pearson's r is indicated. The values of WT and Z allele are labeled by dash lines. Data is presented as mean ± SD, n = 3 biologically independent measurements. **(c)** Full range of molecular variograms for the phenotype landscapes of NE inhibitory activity shown in **Fig. 3c**. Data is presented as mean ± SEM. N of pairwise combinations based on biologically independent measurements of variants is indicated in the Source Data file. The correlation distance range and plateau value of each variogram are indicated. The variograms in the distance range from 0 to 1 are zoomed in **Fig. 3b**. (**d-h**) The residue-based NE inhibitory activity barcode defines three sequence regions at the N-terminal (N1), middle (M2) and C-terminal (C3) regions along the AAT polypeptide sequence that are clustered with variants disrupting AAT activity (**d**). The IVW averaged NE inhibitory activity values for each residue are mapped on the 3D structure of AAT (PDB:3NE4[67]) with the structural regions of N1 (**e**), M2 (**f**), C3 (**g**) and β-sheet B (**h**) highlighted. The N1 region consists of the first five α-helices, hA-E, and two β-strands, s1A and s6B, of AAT **(d-e)**. The M2 region is composed of three β-strands in β-sheet B including s1B, s2B and s3B, two β-strands in β-sheet C including s3C and s4C, and α-helices G and H (hG-H) where the prevalent E288V (S) variant is located that renders partial inactivation of AAT function with reduced load of intracellular polymer relative to AAT-Z (**f**). The dominant E366K (Z) variant allele residue is in the C3 region that includes in addition to β-strand 5A (s5A) and the reaction central loop (RCL), three β-strands at the C-terminal end (**d**; s1C, s4B, s5B; **g**). Though N1, M2 and C3 are separated in the primary sequence of AAT (**d**), they interact with each other through the β-sheet B (**h**).

Supplementary Fig. 7

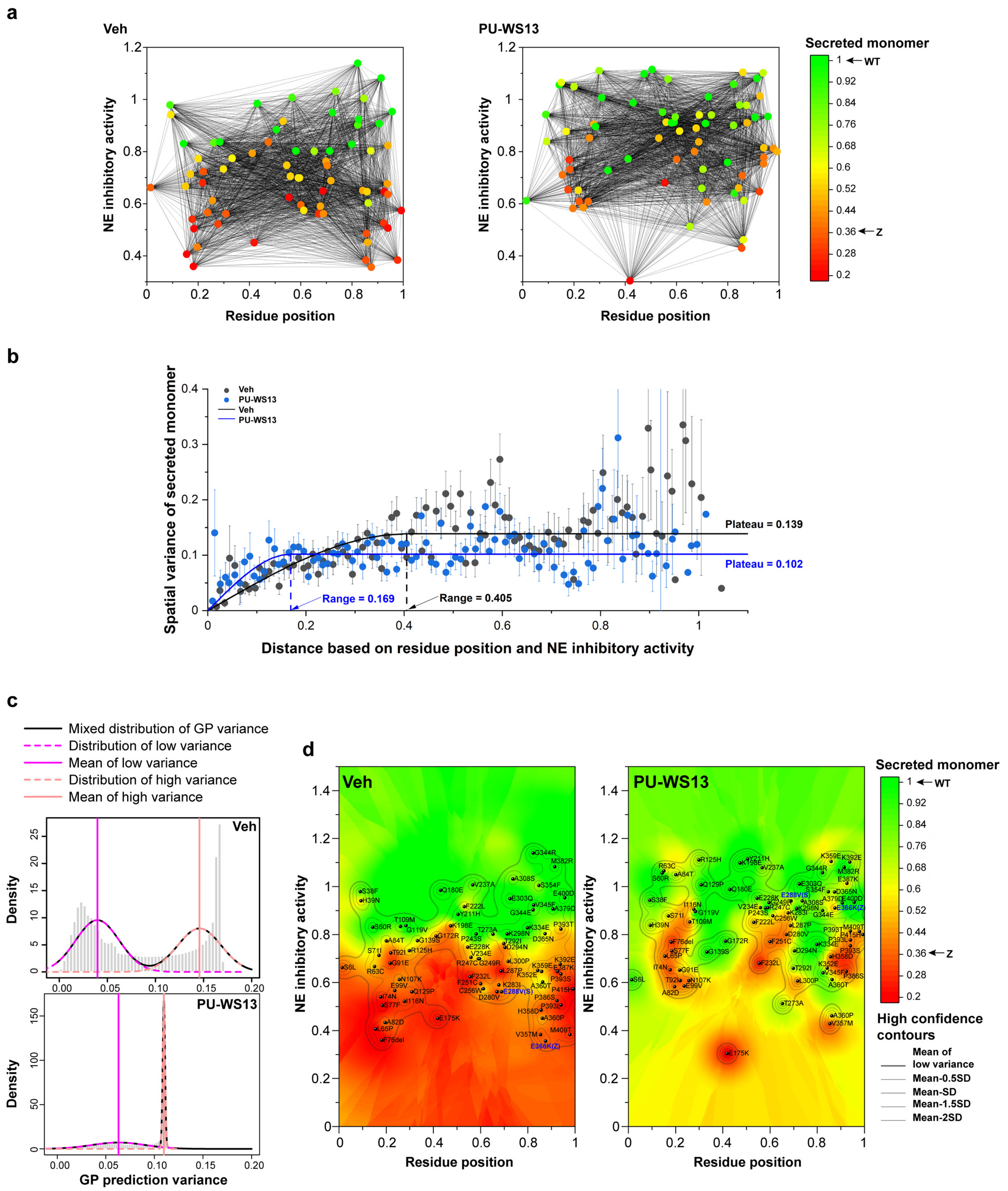

Supplementary Fig. 7. **Phenotype landscapes predicting monomer secretion in response to PU-WS13. (a)** AAT variants are organized by their variant residue position (*x*-axis), NE inhibitory activity (*y*-axis) and secreted monomer (*z*-axis, color scale) in the absence (left panel) or presence (right panel) of PU-WS13. All possible pairwise combinations of variants are illustrated by black lines. **(b)** The spatial variance of secreted monomer and the distance values defined by variant residue positions and NE inhibitory activity for all pairwise combinations are modeled by molecular variograms in the absence (black dots and line) or presence (blue dots and line) of PU-WS13. Data is presented as mean±SEM. n of pairwise combinations based on biologically independent measurements of variants is indicated in the Source Data file. The correlation distance range and plateau value of each variogram are indicated. **(c)** Gaussian mixture model to separate the low *vs* high GP generated variance for each prediction in the absence (upper panel) or presence (lower panel) of PU-WS13. The density of the separated distributions for low variance (magenta dash line) and high variance (pink dash line) are shown. The mixed distribution is illustrated in black curve. The mean of the low variance distribution (magenta line) and high variance distribution (pink line) are indicated. **(d)** Phenotype landscapes generated by GP-based VSP approach linking NE inhibitory activity (*y*-axis) and secreted monomer (*z*-axis, color scale) across the entire AAT polypeptide residue positions (*x*-axis) in the absence (left panel) or presence (right panel) of PU-WS13. The mean of the distribution of low GP variance generated by gaussian mixture model, and the standard deviation (SD) below the mean are illustrated as contours to indicate high confidence predictions. E288V (S) and E366K (Z) are labeled in blue.

Supplementary Fig. 8

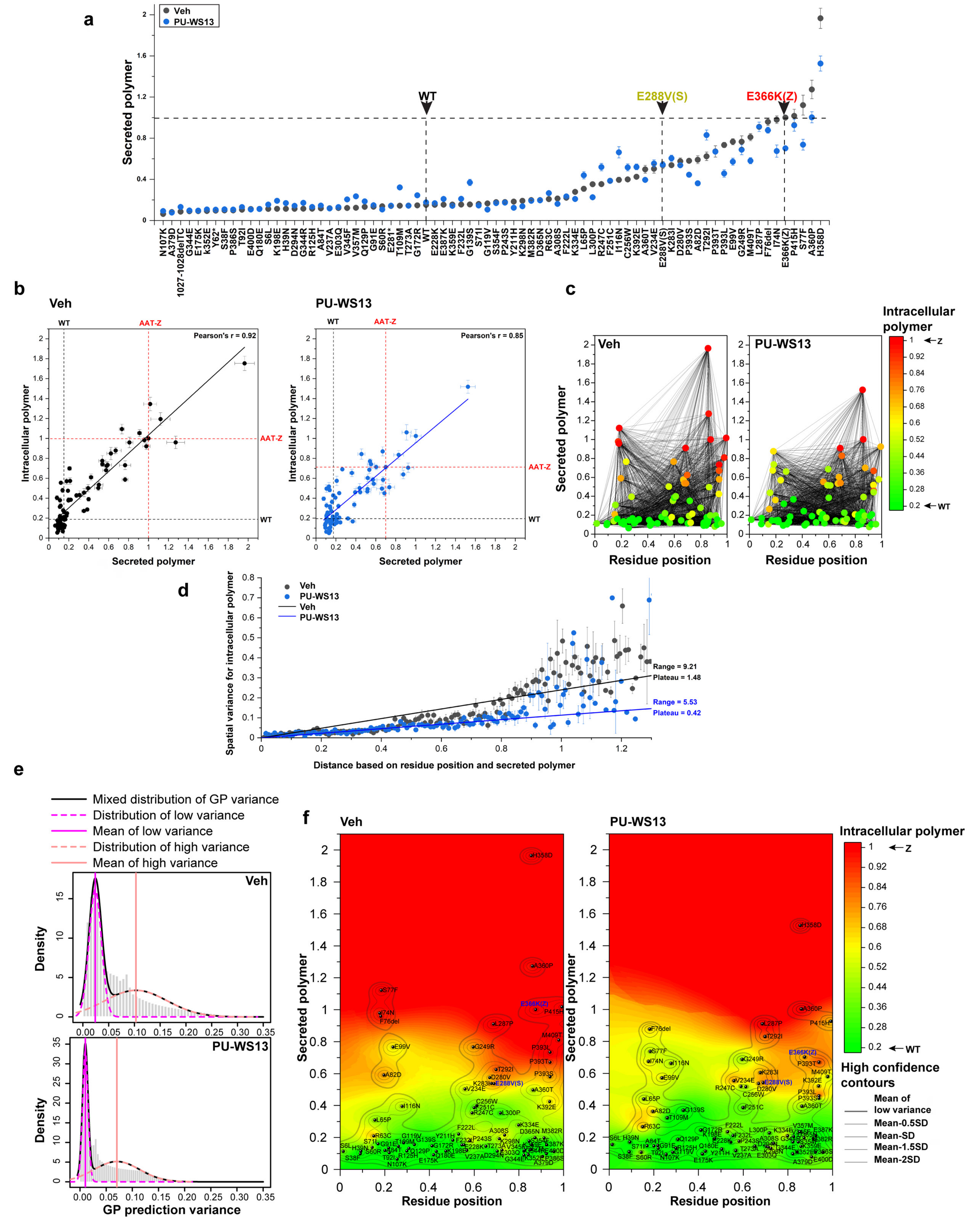

Supplementary Fig. 8. **Phenotype landscapes predicting intracellular polymer in response to PU-WS13. (a)** The responses of AAT variants to PU-WS13 for secreted polymer in Huh7.5^{null} cells. **(b)** Linear correlation between secreted polymer and intracellular polymer of AAT variants in the absence (left panel) or presence (right panel) of PU-WS13. Data is presented as mean±SD; n=3 for secreted polymer and intracellular polymer from 3 biologically independent measurements. Pearson's r is indicated. The values of WT and Z allele are highlighted by dash lines with labels. **(c)** AAT variants are organized by their variant residue position (*x*-axis), secreted polymer (*y*-axis) and intracellular polymer (*z*-axis, color scale) in the absence (left panel) or presence (right panel) of PU-WS13. All possible pairwise combinations of variants are illustrated by black lines. **(d)** The spatial variance of intracellular and the distance values defined by variant residue positions and secreted polymer for all pairwise combinations are modelled by molecular variograms in the absence (black dots and line) or presence (blue dots and line) of PU-WS13. Data is presented as mean±SEM. n of pairwise combinations based on biologically independent measurements is indicated in the Source Data file. The correlation distance range and plateau value of each variogram are indicated. **(e)** Gaussian mixture model to separate the low *vs* high GP generated variance for each prediction in the absence (upper panel) or presence (lower panel) of PU-WS13. The density of the separated distributions for low variance (magenta dash line) and high variance (pink dash line) are shown. The mixed distribution is illustrated in black curve. The mean of the low variance distribution (magenta line) and high variance distribution (pink line) are indicated. **(f)** Phenotype landscapes generated by GP-based VSP approach linking secreted polymer (*y*-axis) and intracellular (*z*-axis, color scale) across the entire AAT polypeptide residue positions (*x*-axis) in the absence (left panel) or presence (right panel) of PU-WS13. The mean of the distribution of low GP variance generated by gaussian mixture model, and the standard deviation (SD) below the mean are illustrated as contours to indicate high confidence predictions. E288V (S) and E366K (Z) are labeled in blue.

Supplementary Fig. 9

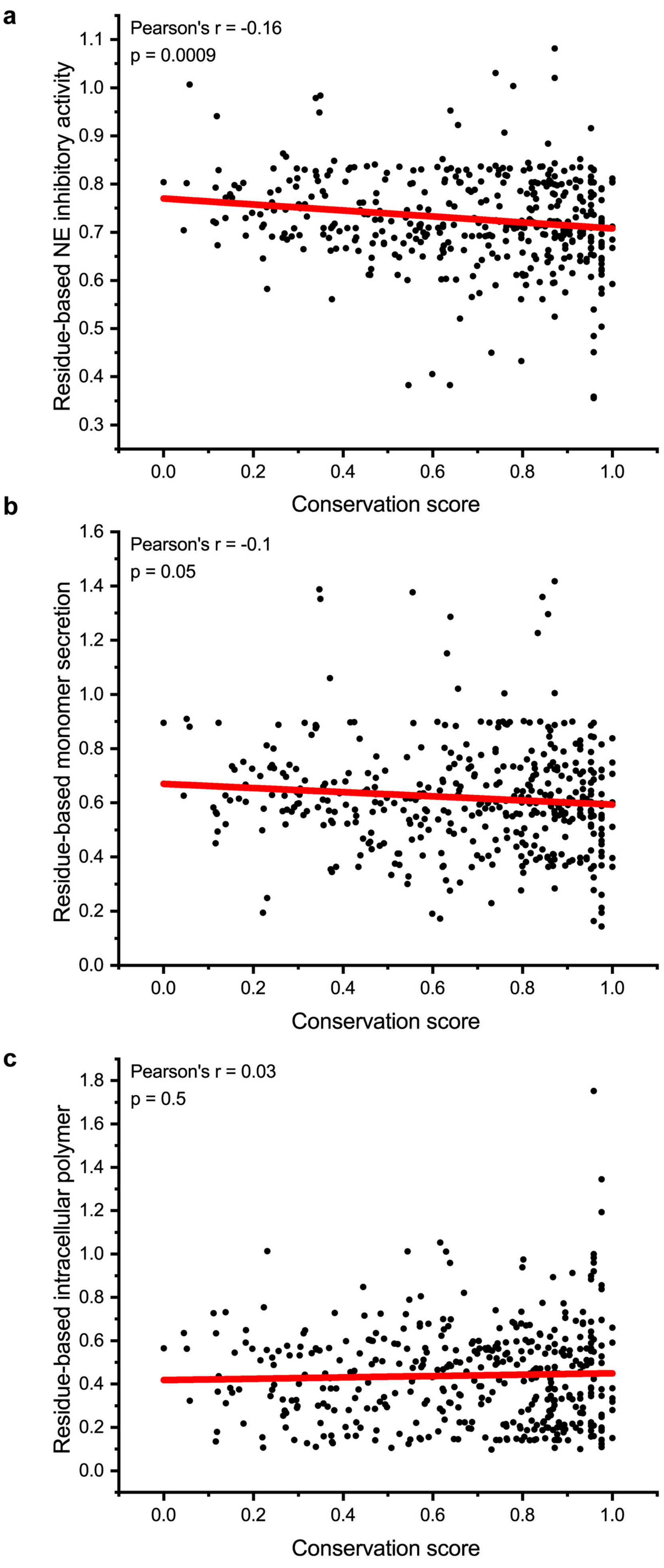

Supplementary Fig. 9. **Comparison of IVW residue-based phenotype value and conservation score.** 109 different AAT sequences from different species were used to generate the conservation scores for each residue using Shannon entropy scores in bio3d package[142]. The conservation score has a weak negative correlation with the SCV-derived NE inhibitory activity score (Pearson's $r = -0.16$, $p = 0.001$) (**a**), suggesting that the conserved residues have a weak trend to confer defective NE inhibitory activity levels. The conservation score has no significant correlation with either monomer secretion (Pearson's $r = -0.1$, $p = 0.05$) (**b**) or intracellular polymer (Pearson's $r = 0.03$, $p = 0.5$) (**c**), indicating that the conservation score does not provide information to understand cellular phenotype responses.

Supplementary Fig. 10

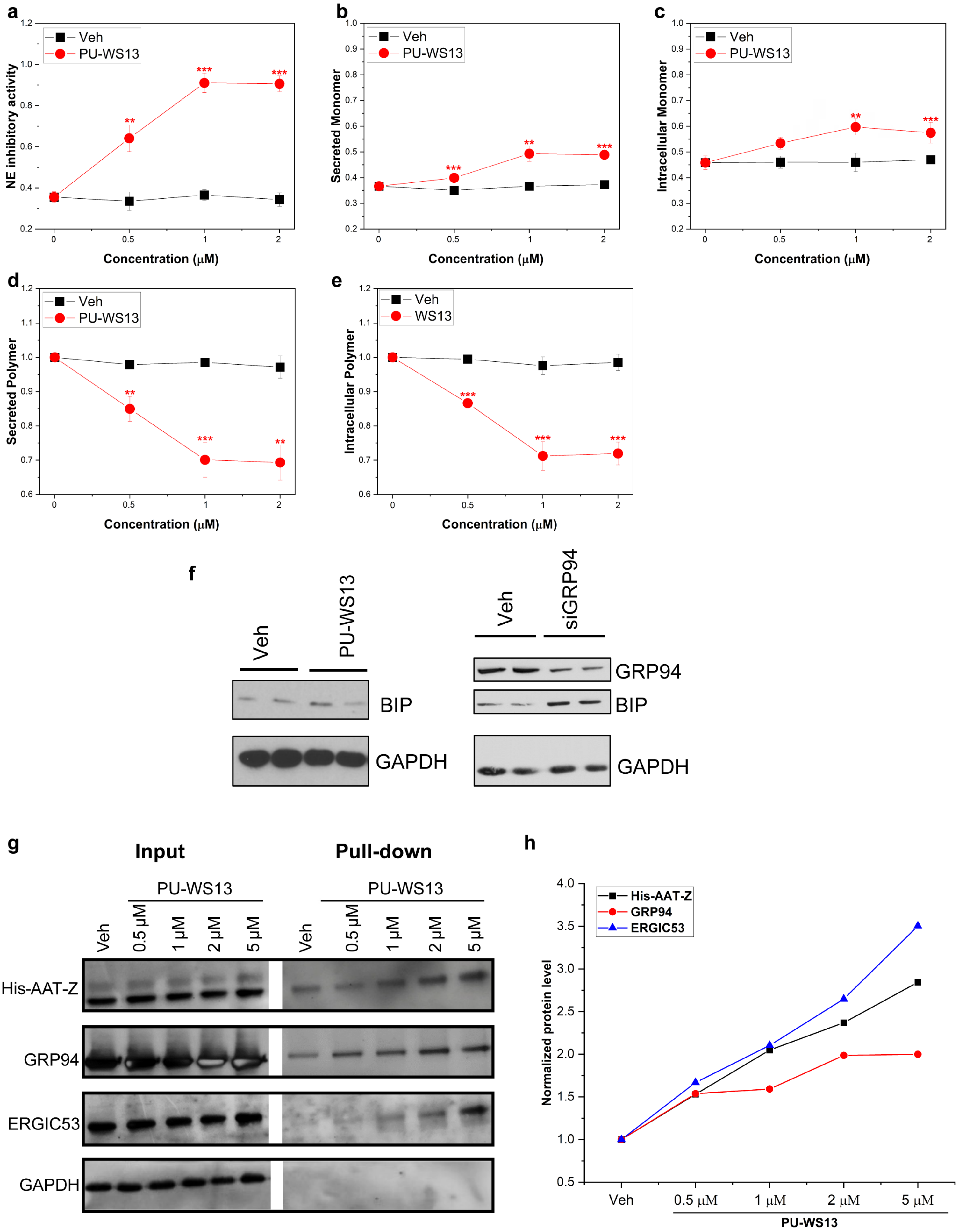

Supplementary Fig. 10. **GRP94 inhibited by PU-WS13 rescues AAT-Z folding, secretion, and function.** (**a**) Huh7.5^{null} cells transiently transfected with AAT-Z were treated by different concentrations of PU-WS13. The neutrophil elastase (NE) inhibitory activity (**a**), secreted monomer levels (**b**), intracellular monomer levels (**c**), secreted polymer levels (**d**), and intracellular polymer levels (**e**) for AAT-Z were measured. Data is presented as mean±SD; n=3 biologically independent measurements. Student's t-test, two tailed; *, $p<0.05$; **, $p<0.01$; ***$p<0.001$; N.S., $p>0.05$. Exact p values were indicated in the Source Data file. (**f**) Cellular BiP levels were detected by immunoblotting in response to PU-WS13 treatment (left panel) or GRP94 siRNA treatment (right panel) in AAT-Z stably transfected IB3 cells. (**g**) Huh7 cells with His-tagged AAT-Z stably expressed were treated with different concentrations of PU-WS13. His-tagged AAT-Z proteins were recovered using HisPur™ Ni-NTA Magnetic Beads. GRP94 and ERGIC53 bound to the recovered with His-tagged AAT-Z (right panel) was detected by immunoblotting. (**h**) The protein level was normalized to vehicle control and plotted relative to PU-WS13 concentration. The recovered level of ERGIC-53 increased relative to that observed for AAT-Z in response to increasing levels of PU-WS13, suggesting increased binding of ERGIC-53 to AAT-Z following PU-WS13 treatment. In contrast, the binding of GRP94 decreased relative to the level recovered AAT-Z in response to increasing PU-WS13, suggesting that PU-WS13 leads to reduced binding between GRP94 and AAT-Z.

Supplementary Fig S11

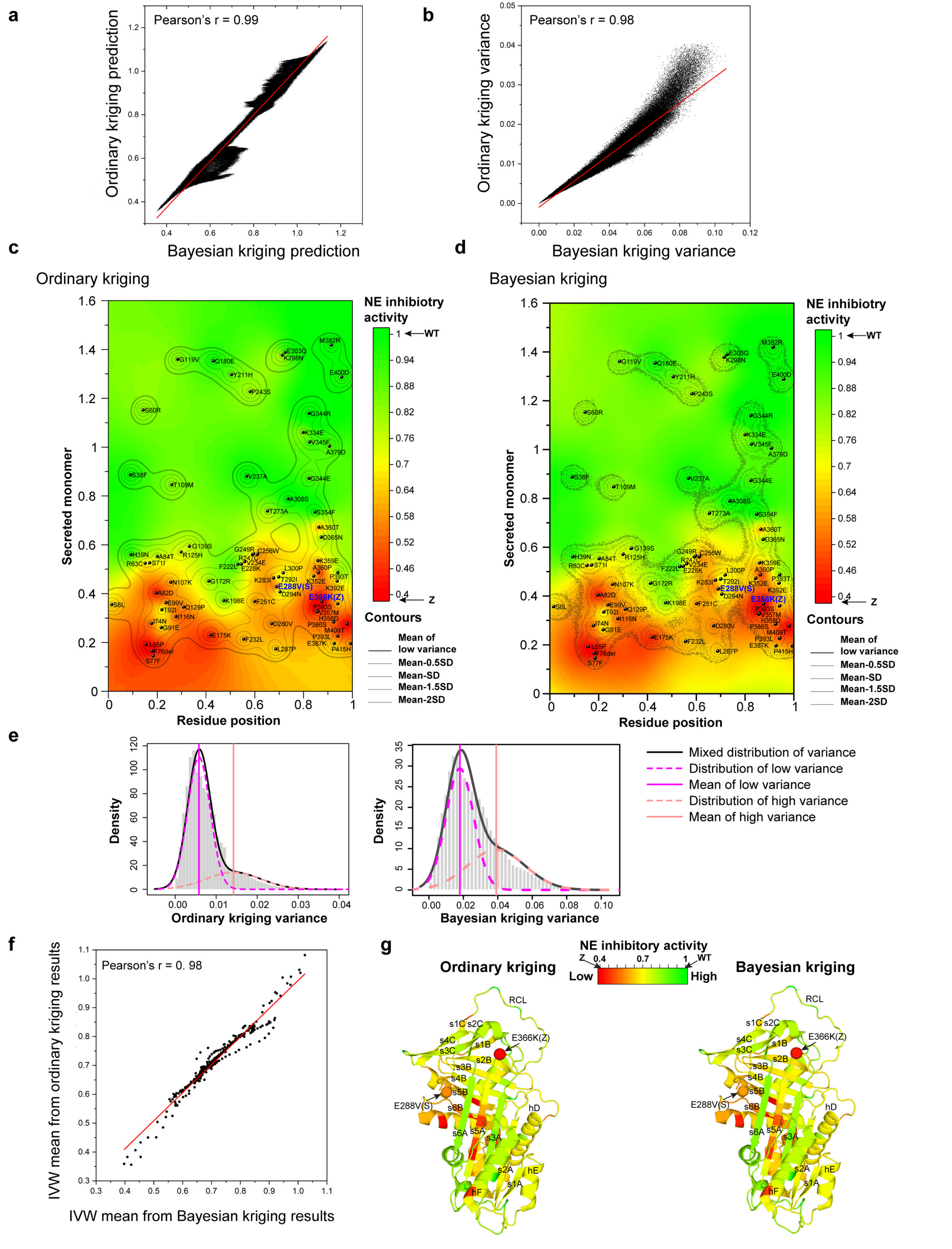

Supplementary Fig. 11. **Comparison between using Ordinary Kriging and Bayesian Kriging for VSP**. (**a**) Correlation of the predictions generated by Ordinary Kriging and Bayesian Kriging (see **Methods**). Pearson's r is indicated. (**b**) Correlation of the prediction variance generated by Ordinary Kriging and Bayesian Kriging. (**c-d**) Phenotype landscapes generated by Ordinary Kriging (**c**) and Bayesian Kriging (**d**). (**e**) Distribution of prediction variance generated by Ordinary Kriging (left) and Bayesian Kriging (right) analyzed by Gaussian mixture model. (**f**) Correlation of the IVW mean generated using Ordinary Kriging and Bayesian Kriging. (**g**) Mapping the IVW mean of NE inhibitory activity generated by Oridinary Kriging (left) and Bayesian Kriging (right) on structure.
