# Supplementary material for: Tracing genetic diversity captures the molecular basis of misfolding disease
Source: Nat Commun. 2024 Apr 18;15:3333. doi: 10.1038/s41467-024-47520-0 (PMC11026414; doi:10.1038/s41467-024-47520-0)
Supplement: Supplementary file 3 — Reporting Summary [file 41467_2024_47520_MOESM3_ESM.pdf]

nature portfolio

Corresponding author(s): William E. Balch

Last updated by author(s): March 05, 2024

Reporting Summary

Nature Portfolio wishes to improve the reproducibility of the work that we publish. This form provides structure for consistency and transparency in reporting. For further information on Nature Portfolio policies, see our Editorial Policies and the Editorial Policy Checklist.

Statistics

For all statistical analyses, confirm that the following items are present in the figure legend, table legend, main text, or Methods section.

n/a	Confirmed	
☐	☒	The exact sample size (*n*) for each experimental group/condition, given as a discrete number and unit of measurement
☐	☒	A statement on whether measurements were taken from distinct samples or whether the same sample was measured repeatedly
☐	☒	The statistical test(s) used AND whether they are one- or two-sided<br>*Only common tests should be described solely by name; describe more complex techniques in the Methods section.*
☐	☒	A description of all covariates tested
☐	☒	A description of any assumptions or corrections, such as tests of normality and adjustment for multiple comparisons
☐	☒	A full description of the statistical parameters including central tendency (e.g. means) or other basic estimates (e.g. regression coefficient) AND variation (e.g. standard deviation) or associated estimates of uncertainty (e.g. confidence intervals)
☐	☒	For null hypothesis testing, the test statistic (e.g. *F*, *t*, *r*) with confidence intervals, effect sizes, degrees of freedom and *P* value noted<br>*Give P values as exact values whenever suitable.*
☐	☒	For Bayesian analysis, information on the choice of priors and Markov chain Monte Carlo settings
☒	☐	For hierarchical and complex designs, identification of the appropriate level for tests and full reporting of outcomes
☐	☒	Estimates of effect sizes (e.g. Cohen's *d*, Pearson's *r*), indicating how they were calculated

Our web collection on statistics for biologists contains articles on many of the points above.

Software and code

Policy information about availability of computer code

Data collection	Immunoblot data were analyzed and collected by by Image J bundled with Java 1.8.0_112 (NIH image);
Data analysis	Structure analysis were conducted by Pymol 1.8.6.0 (Schrodinger, LLC);<br>Student's t-test, Pearson correlation, and one-way ANOVA test of the data were analyzed by Originpro 2021 (Originlab).<br>Gaussian process (GP)-based variation spatial profiling (VSP) analysis was performed in R-studio (Version 2022.10.0) with R (Version 4.2.0).<br>Used Packages include Gstat (Version 2.0.9) ; ggplot2 (Version 3.3.6) ; sp (Version 1.4.7) ; RcolorBrewer (Version 1.1.3) ; Mclust (Version 5.4.9) ; spBayes (Version 0.4-7) ; matrixStates (Version 1.2.0).<br>The input data, R-code scripts and output files for the GP-based analysis have been deposited in the public Mendeley database with DOI: 10.17632/nt59rwz6r6.1. The files are under CC BY 4.0 license.

For manuscripts utilizing custom algorithms or software that are central to the research but not yet described in published literature, software must be made available to editors and reviewers. We strongly encourage code deposition in a community repository (e.g. GitHub). See the Nature Portfolio guidelines for submitting code & software for further information.

Data

Policy information about availability of data

All manuscripts must include a data availability statement. This statement should provide the following information, where applicable:

- Accession codes, unique identifiers, or web links for publicly available datasets
- A description of any restrictions on data availability
- For clinical datasets or third party data, please ensure that the statement adheres to our policy

Source datasets for each figure are provided in the Source Data file. Due to large data size, source data for Fig. 3c-d, Fig. 4a, Supplementary Fig. 7c-d, Supplementary Fig. 8e-f and Supplementary Fig. 11 is included in the Mendeley Data (DOI: 10.17632/nt59rwz6r6.1). PDB files used in this study include 3NE4 [https://doi.org/10.2210/pdb3ne4/pdb], 2D26 [https://doi.org/10.2210/pdb2d26/pdb] and 3T1P [https://doi.org/10.2210/pdb3t1p/pdb].

Research involving human participants, their data, or biological material

Policy information about studies with human participants or human data. See also policy information about sex, gender (identity/presentation), and sexual orientation and race, ethnicity and racism.

Reporting on sex and gender	n/a
Reporting on race, ethnicity, or other socially relevant groupings	n/a
Population characteristics	n/a
Recruitment	n/a
Ethics oversight	n/a

Note that full information on the approval of the study protocol must also be provided in the manuscript.

Field-specific reporting

Please select the one below that is the best fit for your research. If you are not sure, read the appropriate sections before making your selection.

☒ Life sciences ☐ Behavioural & social sciences ☐ Ecological, evolutionary & environmental sciences

For a reference copy of the document with all sections, see nature.com/documents/nr-reporting-summary-flat.pdf

Life sciences study design

All studies must disclose on these points even when the disclosure is negative.

Sample size	The sample size (n) for each experiment is given in the figure legends and in the source data file. For Student's t-test, three biologically independent measurements were performed, except for the native gel experiment shown in Supplementary Fig. 4 e-f, where two biologically independent measurements were performed. For variation spatial profiling (VSP) analysis, our previous studies indicated that the minimum sample size was approximately 50 variants. In the current work, we measured 75 AAT variants and used 72 missense variants as input data for the VSP analysis. The analysis achieved high prediction accuracy as indicated in the paper.
Data exclusions	For the variation spatial profiling analysis, only missense variants were considered as input, as variants causing large protein truncations have very distinct sequence-to-function-to-structure relationships when compared to the missense variants.
Replication	The precise number of repeats (n) are indicated in the figure legend and Source Data file for each experiment.
Randomization	There is no clinical research in this study. Randomization is not applied to this study.
Blinding	There is no clinical research in this study. Blinding is not applied to this study.

Reporting for specific materials, systems and methods

We require information from authors about some types of materials, experimental systems and methods used in many studies. Here, indicate whether each material, system or method listed is relevant to your study. If you are not sure if a list item applies to your research, read the appropriate section before selecting a response.

Materials & experimental systems

n/a	Involved in the study
☐	☒ Antibodies
☐	☒ Eukaryotic cell lines
☒	☐ Palaeontology and archaeology
☒	☐ Animals and other organisms
☒	☐ Clinical data
☒	☐ Dual use research of concern
☒	☐ Plants

Methods

n/a	Involved in the study
☒	☐ ChIP-seq
☒	☐ Flow cytometry
☒	☐ MRI-based neuroimaging

Antibodies

Antibodies used

The goat anti-human AAT polyclonal antibody (80A) was purchased originally from ICL. Inc (Anaheim, CA) (Cat # GCYT-80A, 1:1000 dilution).
The mouse anti-human AAT monoclonal antibody 16F8 used in this paper was generated in house by Scripps Research Antibody Development and Production Core (1:2000 dilution).
The mouse anti-human AAT monoclonal antibody 2C1 was purchased from Hycult Biotech (Wayne, PA) (Cat # HM2289, 1:1000 dilution).
The GRP94 antibody was purchased from Abcam (Waltham, MA) (Cat # ab238126, 1:1000 dilution).
The BiP/GRP78 antibody was purchase from Abcam (Waltham, MA) (Cat # ab108615, 1: 2000 dilution).
The ERGIC53 antibody was purchased from Proteintech Inc (Rosemont, IL) (Cat # 13364-1-AP, 1:20000 dilution)
Anti-His-tag antibody, Clone 1B7G5, was purchased from Proteintech Inc. (Cat # 50-173-6447, 1:5000 dilution).
GAPDH antibody was purchased from Abcam (Cat # ab8245, 1:5000 dilution).
Secondary antibody the goat anti-mouse HRP antibody (Cat # 32230; RRID: AB_1965958, 1:5000 dilution), goat anti-rabbit HRP antibody (Cat# 32260; RRID: AB_1965959, 1:5000 dilution) and mouse anti-goat HRP antibody (Cat#31400; RRID: AB_228370, 1:10000 dilution) were purchased from Thermo Fisher Scientific (Waltham, MA).

Validation

The goat anti-human AAT polyclonal antibody (80A) (ICL. Inc, Cat # GCYT-80A) has been validated as manufacturer's ELISA protocol: https://www.icllab.com/human-alpha-1-antitrypsin-elisa-kit-e-80a1t.html
The mouse anti-human AAT monoclonal antibody 16F8 was validated by purified AAT proteins and described in supplementary figure.1a.
The mouse anti-human AAT monoclonal antibody 2C1 from Hycult Biotech (Cat # HM2289) was tested positively in ELISA assay as the manufacturer's protocol: https://www.hycultbiotech.com/product/alpha-1-antitrypsin-human-mab-2c1/
The GRP94 antibody from Abcam (Cat # ab238126) was validated in Huh7.5 null cells by WB as the manufacturer's protocol: https://www.abcam.com/en-hk/products/primary-antibodies/grp94-antibody-epr22847-50-ab238126
The BIP/GRP89 antibody from Abcam (Cat # ab108615) was tested in Huh7.5 null cells by WB as the manufacturer's protocol: https://www.abcam.com/en-hk/products/primary-antibodies/grp78-bip-antibody-epr40412-ab108615#application=wb
The ERGIC53 antibody from Proteintech Inc (Cat # 13364-1-AP) was validated by WB as the manufacturer's protocol: https://www.ptglab.com/products/LMAN1-Antibody-13364-1-AP.htm
Anti-His-tag antibody, Clone 1B7G, from Proteintech Inc (Cat # 50-173-6447) was tested by WB according to manufacturer's protocol: https://www.fishersci.com/shop/products/his-tag-mouse-anti-tag-clone-1b7g5-proteintech/501736447
GAPDH antibody from Abcam (Cat # ab8245) was validated as manufacturer's protocol by WB: https://www.abcam.com/en-hk/products/primary-antibodies/gapdh-antibody-6c5-loading-control-ab8245
Secondary antibodies were all from Thermo Fisher Scientific and tested positive according to manufacturer's protocols: the goat anti-mouse HRP antibody (Cat # 32230) https://www.thermofisher.com/antibody/product/Goat-anti-Mouse-IgG-H-L-Poly-HRP-Secondary-Antibody-Polyclonal/32230;
the mouse anti-rabbit goat HRP antibody (Cat # 32260): https://www.thermofisher.com/antibody/product/Goat-anti-Rabbit-IgG-H-L-Poly-HRP-Secondary-Antibody-Polyclonal/32260
the mouse anti-goat HRP antibody (Cat # 31400): https://www.thermofisher.com/antibody/product/Mouse-anti-Goat-IgG-H-L-Cross-Adsorbed-Secondary-Antibody-Polyclonal/31400

Eukaryotic cell lines

Policy information about cell lines and Sex and Gender in Research

Cell line source(s)

IB3 cells stably transfected with AAT-Z, which were provided by T. Flotte, University of Massachusetts Medical School, Worcester, MA.
Huh7 cells with His-tagged Z-AAT stably expressed were provided by T. Flotte, University of Massachusetts Medical School, Worcester, MA.
Huh7.5 AAT knockout (KO) cells (Huh7.5null), which were provided by Mark Brantly, University of Florida College of Medicine, Gainesville, FL.
Human AAT-ZZ hiPSC cells were purchased from DefiniGEN (Cambridge, UK).

Authentication

Human AAT-ZZ hiPSC cells were purchased directly from DefiniGEN (Cambridge, UK) and verified by AAT expression using AAT antibody. IB3 cells stably transfected with AAT-Z, Huh7 cells stably transfected with His-AAT-Z, and Huh7.5 cells with AAT knockout were all verified using AAT antibody.

Mycoplasma contamination	No mycoplasma contamination was found in the cell lines used in this study.
Commonly misidentified lines<br>(See ICLAC register)	Commonly misidentified cell lines were not used in this study.
